# Supplementary material for: Temporal and spatial differences in the vaginal microbiome of Chinese healthy women
Source: PeerJ. 2023 Dec 1;11:e16438. doi: 10.7717/peerj.16438 (PMC10695111; doi:10.7717/peerj.16438)
Supplement: Supplemental Information 1 [file peerj-11-16438-s001.docx]

**Supplementary**

**Results and discussion**

We compared the composition of vaginal microbiome at three taxonomic levels in cervical orifice and mid-vagina of healthy women during different periods and found that the composition of vaginal microbime in two samples was significantly different from the others (Supplementary fig. A, B). The relatively high abundance of pathogenic microbial genera and species were found in three periods of sample NO. 9 and period A of sample NO. 14, including Gardnerella and *G. vaginalis* (Supplementary fig. C). Besides, Prevotella and the corresponding species *Prevotella intermedia* were found relatively more often in individual No. 14 during period A. The above results explained the large bias in the LEfSe we subsequently performed (Supplementary fig. D). At three taxonomic levels, significant outliers occurred in samples of No. 9 and No. 14.

*G. vaginalis* has mild symptoms and is often associated with frequent sexual activity and poor hygiene. We found that individual No. 9 had *G. vaginalis* in the mid-vagina throughout the menstrual cycle, and we speculated that this phenomenon occurred due to volunteer hygiene habits. In individual NO. 14, the relative abundance of *G. vaginalis* was high only during period A, while the relative abundance of *Lactobacillus* was absolutely dominant during period B and C, indicating that the opportunistic pathogens was gradually reduced under the protection of *Lactobacillus*.
